# Biomonitoring of Heavy Metal Pollution Using Acanthocephalans Parasite in Ecosystem: An Updated Overview

**DOI:** 10.3390/ani10050811

**Published:** 2020-05-07

**Authors:** El-Sayed E. Mehana, Asmaa F. Khafaga, Samar S. Elblehi, Mohamed E. Abd El-Hack, Mohammed A.E. Naiel, May Bin-Jumah, Sarah I. Othman, Ahmed A. Allam

**Affiliations:** 1 Department of Pathology, Faculty of Veterinary Medicine, Alexandria University, Edfina 22758, Egypt; elsayedhamouda@gmail.com (E.-S.E.M.); Samarsaad30@yahoo.com (S.S.E.); 2 Poultry Department, Faculty of Agriculture and Zagazig University, Zagazig 44511, Egypt; dr.mohamed.e.abdalhaq@gmail.com; 3 Department of Animal Production, Faculty of Agriculture, Zagazig University, Zagazig 44511, Egypt; 4 Department of Biology, College of Science, Princess Nourah bint Abdulrahman University, Riyadh 11474, Saudi Arabia; may_binjumah@outlook.com (M.B.-J.); sialothman@pnu.edu.sa (S.I.O.); 5 Department of Zoology, Faculty of Science, Beni-suef University, Beni-suef 65211, Egypt; allam1081981@yahoo.com

**Keywords:** heavy metals, fish, parasite, bioaccumulation

## Abstract

**Simple Summary:**

The environment receives different sources of pollutants, resulting from human industrial pollution as well as activities. This review updates and focuses the light on the relation between the toxicity of heavy metals resulting from bioaccumulation in fish and the parasite bioindication role and its infestation.

**Abstract:**

As a result of the global industrial revolution, contamination of the ecosystem by heavy metals has given rise to one of the most important ecological and organismic problems, particularly human, early developmental stages of fish and animal life. The bioaccumulation of heavy metals in fish tissues can be influenced by several factors, including metal concentration, exposure time, method of metal ingestion and environmental conditions, such as water temperature. Upon recognizing the danger of contamination from heavy metals and the effects on the ecosystem that support life on earth, new ways of monitoring and controlling this pollution, besides the practical ones, had to be found. Diverse living organisms, such as insects, fish, planktons, livestock and bacteria can be used as bioindicators for monitoring the health of the natural ecosystem of the environment. Parasites have attracted intense interest from parasitic ecologists, because of the variety of different ways in which they respond to human activity contamination as prospective indices of environmental quality. Previous studies showed that fish intestinal helminths might consider potential bioindicators for heavy metal contamination in aquatic creatures. In particular, cestodes and acanthocephalans have an increased capacity to accumulate heavy metals, where, for example, metal concentrations in acanthocephalans were several thousand times higher than in host tissues. On the other hand, parasitic infestation in fish could induce significant damage to the physiologic and biochemical processes inside the fish body. It may encourage serious impairment to the physiologic and general health status of fish. Thus, this review aimed to highlight the role of heavy metal accumulation, fish histopathological signs and parasitic infestation in monitoring the ecosystem pollutions and their relationship with each other.

## 1. Introduction

For the time being, the environment receives different sources of pollutants, resulting from human industrial pollution as well as activities [1,2]. Heavy metals such as lead, mercury, cadmium, etc., naturally occur in the deep layers of the earth and are present in the soils, rocks and sediments with high concentrations [3]. Additionally, geological weathering, anthropogenic emissions from crude mines, getting metals from the underground mines and mining activities would lead to an increase in the concentrations of heavy metals in the surrounding environment and may pollute water [4]. Various heavy metals cannot be degraded and persist in the environment. Consequently, they accumulate in lake sediments (estuarine or marine) [5]. Biologically, heavy metals are classified into essential metals (e.g., Fe, Ni, Cu and Zn) which are essential for the fish metabolism and non-essential metals (e.g., Pb, Hg and Cd), which are toxic even in traces, and have unknown functions in the biological systems [6,7]. Fish are particularly vulnerable and heavily exposed to pollutants due to feeding and living in aquatic ecosystems, because they cannot avoid pollutant harmful effects [8]. Heavy metals enter fish by direct absorption from water through their gills and skin, or by ingestion of contaminated food [9]. The metals then enter the bloodstream of the fish and gradually accumulate in their tissues, particularly in the liver, where they are bio-transformed and excreted or passed over to consumers through the food chain [10]. Hence, heavy metals, as well as parasites, induce significant damage to the physiology and biochemical processes and may induce serious impairment to the physiology and the health status of fish [11]. Besides, it can alternate the normal biochemical reactions and induce deleterious effects such as inhibition of growth, impaired respiration, oxygen consumption and the failure of reproduction and regeneration of different tissues [12]. However, the deleterious effects of heavy metal exposures on living organisms need to be more elucidated [13].

A new approach is chosen to visualize ecosystem health by using some living organisms as bioindicators [14]. In this aspect, macrophytes, phytoplankton, invertebrates, and fish are widely used as bioindicators for heavy metals pollution [15,16]. Fish parasites are considered very sensitive to heavy metal pollution, as they not only accumulate toxicants in their tissues, but they also exert a physiological response to it [17]. Parasites can be used either as effect indicators or as accumulation indicators, because of the variety of ways in which they respond to anthropogenic pollution [18,19]. Accumulation indicators are organisms that can concentrate certain substances in their tissues to levels significantly higher than those in the ambient. So, intestinal helminths parasites affecting fish can be used in the biomonitoring of heavy metal pollution in the aquatic environment [20]. Indeed, intestinal parasites of fish as acanthocephalans are thorny-headed worms that can accumulate higher concentrations levels of heavy metals than those accumulated in the host tissues [21,22]. In this aspect, helminths parasites, especially intestinal ones (trematodes, nematodes, cestodes, and acanthocephalans) are used as biological indicators for heavy metal pollution in the aquatic environment [20]. According to the degree of heavy metals bioaccumulation, Acanthocephalans and Cestodes are considered a good bioindicator, because they accumulate higher heavy metal concentrations, especially the toxic types in their tissues [23]. Meanwhile, parasitic nematodes can accumulate lower concentrations of heavy metals in their bodies and this ability appears to be variable and depends on nematode species [24,25]. Additionally, trematodes of fish [26] tend to accumulate a lower level of some metals in their tissues than cestodes and acanthocephalans [23]. On the other hand, comprehensive evidence has been provided to recognize that the presence of nematode (anisakid) larvae in the body cavity of many economically fish species may affect the processing of fish and may also have implications for public health [27]. Additionally, certain migratory zooplankton, especially marine ostracods and cirolanid isopods, attack live fish at night, some of which can cause major damage to commercial fish [28]. Therefore, this review aimed at gathering, updating, and shedding light on the relationship between the heavy metal toxicity and bioaccumulation in fish and the parasitic infestation.

## 2. Heavy Metals Pollution in Fish

### 2.1. Bioaccumulation

The main threats for fish consumers are associated with exposure to cadmium, mercury, arsenic, and lead [29]. For human beings, there are several different sources of heavy metal pollution such as rechargeable nickel-cadmium batteries and cigarette smoking, which is considered as the major source for cadmium exposure, inducing serious effects such as renal damage and bone fracture [30]. However, humans could be exposed to mercury through food, fish and using or breaking products containing mercury [31]. Bioaccumulated mercury can induce neuronal damage for fish consumers [32]. Besides, consumers are exposed to lead metal from the air, paint, food, and petrol [33]. Gastrointestinal disturbances in children, as well as nephrotoxicity and neurotoxicity in adults, are the common adverse effects of lead toxicosis in humans [34]. Additionally, exposure to arsenic usually occurs via drinking water that caused skin diseases, hyperkeratosis, and melanosis [35]. Although there are many sources for heavy metal contamination, they finally reached the fish, causing dangerous effects on fish as well as fish consumers. These exposure sources are usually increased due to the development of human activities, increased industrialization, and waste discharge into the fish environments [36,37,38]. The most famous types of heavy metal causing pollution for fish are mercury, copper, cadmium, lead, zinc, chromium, manganese, and iron [39]. Whereas some of these metals are vital for fish health at recommended levels [40], for others the induced toxicity usually begins when they exceed these levels [41], as illustrated in Table 1.

Heavy metals are sensitive biomarkers for both fish health and the ecology of water bodies [48]. Indeed, heavy metals are a natural trace constituent in the aquatic environment; they are an essential cofactor for many of the enzymes that are needed for many metabolic activities [49]. The bioaccumulation of heavy metals and parasites burden in Nile tilapia is considered a proxy of both water quality and ecology [50,51]. Riad et al. [52] examined the physicochemical and bacteriological parameter in the Nasser lake in Egypt (60 fish samples and 36 water samples); they concluded that the concentration of heavy metals (copper, zinc, lead and iron) in water was within the permissible limits [52]. The main source of most environmental pollution is industrial waste that has been released into the water bodies, where it has been transferred into the aquatic system like fish. However, unfortunately, their levels have been elevated because of the industrial wastes, agricultural and mining activities, and the geochemical structure [53]. When the concentration of heavy metals (in water) exceeds the permissible limits (according to both WHO/FEPA), it will cause a serious threat to human health due to their ability to bioaccumulate in fish tissues [48]. Therefore, they could prove a useful tool in biomonitoring or toxicity assessment studies.

**Figure 1:**
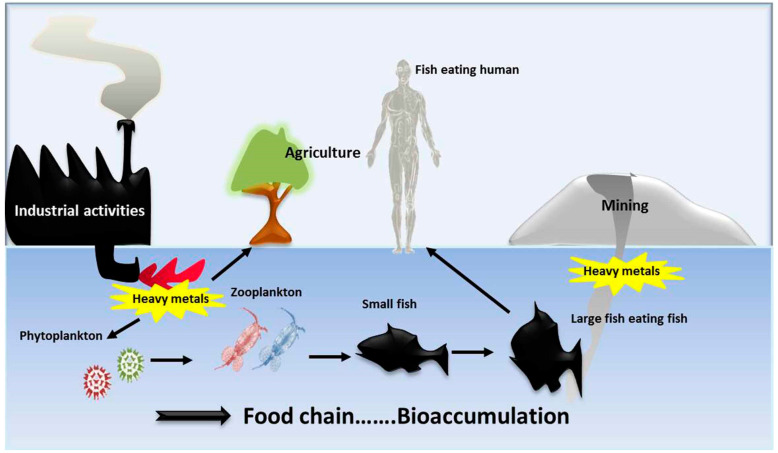
The bioaccumulation of heavy metals due to mining and industrial activities, and its effect on the aquatic food chain.

The absorption and accumulation of different types of heavy metals are representing a big hazardous for the fish ecosystem [54]. Atobatele and Olutona [55] studied the differential accumulation of heavy metals (cadmium, mercury, and lead) in the liver, kidneys, and gills of seven species of water fish, as bioindicators for the quality of the fish ecosystem. The recorded levels were 0.001–0.100 ppm, 0.000–0.067 ppm, and 0.001–0.125 ppm for cadmium, mercury, and lead; respectively. In general, heavy metals enter the aquatic environment mainly by anthropogenic sources (human and domestic); these metals tend to accumulate mainly in the liver as an edible tissues, kidneys, muscle, heart, and brain of the infected fish [56]. The accumulation of heavy metals in fish tissues is influenced by several extrinsic factors such as metal concentration, exposure period, way of metal uptake, and environmental parameters as water temperature; or intrinsic factors such as size and age [57]. Copper is necessary for fish normal growth and metabolism. Most of the metals and other contaminants are also taken up by the host’s digestive system and even by environmental conditions; the host’s physiology also plays an important role in the accumulation mechanism [58,59]. Recently, different types of heavy metals (Zn, Cu, Cr, Pb, Cd, and Fe) were assessed in the Chad Bath region ecosystem, using a spectrophotometer. All the previously mentioned metals were in levels higher than the permissible limits (according to the international standards); the increased metals concentrations in water were considered a good indicator for fish ecosystem pollution [57,60,61]. In another way, Kim et al. [62] reported that the Fish based-diets had a significantly higher level of arsenic, cadmium and mercury, relative to poultry or red meat diets. In general, the ability of heavy metals to bioaccumulate and biomagnifying and difficulty to be eliminated from the body by the ordinary metabolic activities makes them one of the most dangerous sources of chemical water pollution to fish, causing big losses to fish and effects on the fish consumers [63]. The bioaccumulation of heavy metals due to mining and industrial activities and its effect on the aquatic food chain is illustrated in Figure 1.

### 2.2. Histopathology

Histopathology supplies a faster way to detect the effects of irritants (heavy metals for example) and pathogens (as protozoan parasites) in different fish organs, and it can be considered as the indicator for many abnormal conditions for the fish ecosystem (environment) [8,64]. However, the pathology of heavy metals varies according to the exposure period, metals concentration, and genetic susceptibility. Therefore, it cannot be considered a real diagnostic tool. Zeitoun and Mehana [54] and Abalaka [65] studied the bioaccumulation and the histopathologic effects of heavy metals “Cd, Pb and Zn” in water and fish (*Auchenoglanis occidentalis*), from Tiga dam in Nigeria. The characteristic pathological and bio-cumulative alterations were shown in gills, liver, and kidneys. Lesions in gills were in the form of lamellar epithelial hyperplasia, fusion, necrosis, and detachments. However, hepatic and renal alterations were in the form of vacuolar and hydropic degeneration of both hepatocytes and renal epithelial cells, that were finally detached and necrotized. In addition, mononuclear cells infiltrations and hemorrhage were also a characteristic histopathologic picture in liver and kidney tissues. Additionally, Saini [66] documented significant histopathological lesions such as nuclear cell infiltration, hepatic parenchyma degeneration and hepatocyte deformation caused by heavy metal (arsenic), in *L. rohita* liver, captured from natural Punjab freshwaters. Specific histopathological changes such as “enlargement of the renal tubules, desquamation of the epithelial lining, edema, dilation of the renal tubules, severe necrosis, pyknotic nuclei, vacuolization, disorganized blood capillaries in glomerulus” have been caused in the *Channa punctatus* kidneys following exposure to sublethal zinc concentration [67]. Likewise, Dhevakrishnan and Zaman [68] investigated that Cauvery river contaminants (mercury, lead, copper and cadmium) have also caused many histopathological alterations in *L. Rohita* muscle tissue, which included muscle bundle shortening, severe intramuscular oedema and muscle bundle necrosis.

In another study, chub (freshwater fish) was caught from different heavy metals polluted sites and examined for the histopathologic lesions in the liver and gills. Results revealed that the main pathologic alterations in gills lamellae are epithelial hyperplasia, hypertrophy, fusion, shortening, lifting, congestion, and necrosis. In addition, the lamellar aneurysm was also characteristic. The hepatic lesions were in the form of hepatocytes degeneration and necrosis, with a congested central vein, mononuclear infiltration, and melanomacrophage center aggregations [11,69]. Recently, Mshelbwala et al. [70] experimented with detecting the concentrations of Zn, Pb, Ni, Cu, Mn, Fe, Cr and Cd in the edible parts of freshwater mussels, as well as in the water and sediments of the Kabul River, Khyber Pakhtunkhwa, Pakistan; the histopathologic alterations in gills and GIT were used as biomarkers in the biomonitoring of heavy metals pollution in the aquatic ecosystems. The authors concluded that the pathologic changes were represented by branchial and intestinal epithelial cells vacuolization, intestinal lipofuscinosis, lamellar necrosis, and mononuclear cell infiltration.

## 3. Parasitic Infestation in Fish

Some of the fish parasites such as parasitic crustacean are destructive organisms that injured the fish’s health; the extent of fish damage is dependent on the role played by the fish in the parasite cycles [71]. Usually, the intermediate host suffered more than the definitive hosts. Additionally, the damaging effects of the parasites might occur indirectly by uptaking the fish nutrients [72]. Fish parasites represent a major part of aquatic biodiversity, and fish become affected either directly through the environment or indirectly through their respective hosts [73]. Recently, fish-parasite-heavy metals are considered as an effective monitoring system to evaluate the quality of the environmental fish ecosystem, where the parasites can indicate different pollutants in fish environments, such as heavy metals and sewage pollution. Herein, fish parasites could be used as a biological indicator, to illustrate the ecology of the infected hosts including migration, feeding, and population culture. Several classes of parasites such as Monogenea, Rhabditophora, Cestodes, or Hexanauplia could infect the freshwater or marine water fish. Infected fish tissues revealed severe histologic cellular responses, with different degrees of severity correlated with the severity of the parasitic infestation. 

Feist and Longshaw [74] studied the effect of monogenea induced necrosis of gills, kidneys, and liver with induced osmoregulatory disturbances and fish death. Meanwhile, Mohammadi et al. [75] conducted a pathologic study in two aquarium species (Oscar and Discus), to investigate the harmful effects of parasites. Tissue samples were collected from gills and skin. Four parasite species were isolated (*Dactylogyrus* spp., *Trichodina*, *Gyrodactylus* spp., and *Ichthyophthrius multifillis*). The detected histologic lesions were in the form of lamellar hyperplasia, fusion, and necrosis of the epithelial cells of the gills and skin epidermis. Besides, lamellar aneurysm, edema, purulent bronchitis, and dermatitis were also noted. In another study, Nahavandinejad et al. [76] evaluated one hundred freshwater ornamental fish in Iran for parasitic infestation through a histopathologic monitoring program; results revealed that fish were infested with *Eimeria* spp., *Cryptosporidium* spp., *Tetrahymena*, *Giardia*, and *Myxobolous*. The use of parasites as biological indicators for heavy metals bioaccumulation is presented in Table 2.

## 4. Relationship between Heavy Metals Pollution and Parasite Infestation in Fish

**Figure 2:**
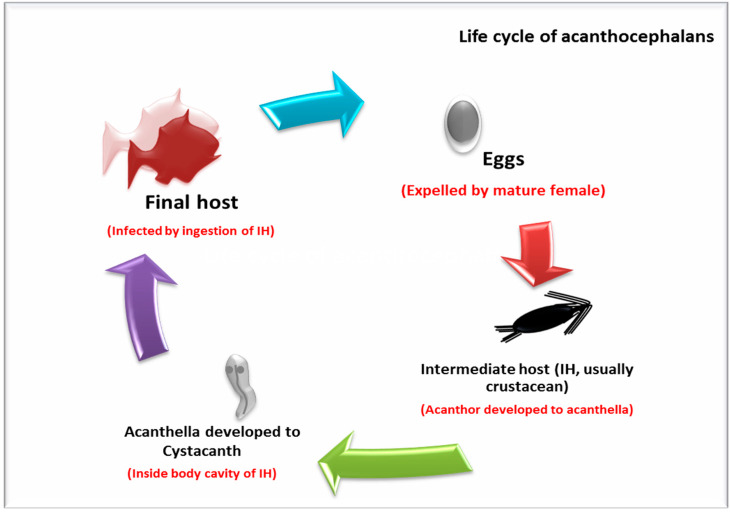
Life cycle of acanthocephalan.

Several types of parasites (such as intestinal Cestodes and Acanthocephala) are used as biological indicators to detect the quality of fish environmental ecologies, due to their higher response to the different types of anthropogenic pollutants in the water. In other words, it is easy for the parasite to detect the chemical state of the water, which is used as an important indicator for heavy metals pollution in both freshwater and saltwater [87]. Interestingly, it has been reported that the concentration of heavy metals was higher in the intestinal parasites acanthocephalan several thousand times in comparison to the fish tissues (liver, kidneys, gills, and muscles) (life cycle of acanthocephalan is illustrated in Figure 2). The presence of the bile acids in the lumen of fish resulted in the formation of organic-metallic complexes, which are easily absorbed by the worms due to lipophilicity [18,87,88]. Malek et al. [79] concluded that the concentration of lead and cadmium in Cestoda “*Paraotigmatobothrium* spp. and *Anthrobothrium* spp.” were higher than their levels in liver, kidneys, and gonads of shark collected from Iranian coastal water. This finding has strongly supported the theory that the helminths parasites are extremely sensitive bioindicators that may serve as an early warning, particularly in the sensitive low-level environmental threats. Therefore, they may also have beneficial effects on the health of their host by acting as heavy metal filters [79].

On the other hand, harmful interactions between parasites and heavy metals pollution were reported, where the parasite enhances the toxic effects of heavy metals by interfering with the fish-protection mechanism. As well, fish parasites had extensively negative effects on the physiological homeostasis of fish. The combination of the two stressors had adverse negative impacts on both fish and fish consumers [89].

The majority of fish serve as an intermediate host for many parasites, which reduces the food value of fish and causes mass mortality [90]. Bayoumy et al. [91] concluded that several species of parasite, including metazoan parasite, crustaceans, digenean, and Monogenea were detected in different tissues of Nile tilapia fish. Monogenea showed highly antagonistic action with chromium, iron, and nickel. Meanwhile, the crustacean parasites showed the highest significant positive correlation with zinc and selenium. In another investigation, Bayoumy et al. [91] isolated external and internal metazoan parasites from three fish species in the Arabian Gulf of Dammam coast in different environmental weather. The rate of parasitism was higher in spring and summer in comparison to the winter season. In this study, crustacean parasites, Digenea, and Monogenea showed a highly significant positive correlation with Zn and Se. Moreover, Tellez and Merchant [86] investigate the exposure of both fish and alligators to parasites and heavy environmental pollution with heavy metals, using the monitoring programs as a useful tool to detect metal concentrations and exposure. Parasites, particularly intestinal trematodes, collectively accumulated higher levels of Se, Cu, As and Zn; they acted like sensitive bioindicators for heavy metals pollution [92].

In 2016, Bamidele and Kuton [93] used *Parachanna obscura* and *Clarias gariepinus* fish to evaluate the bioaccumulation of heavy metals in fish tissues and parasites (intestinal nematodes), as an indicator for heavy metals (Cu, Cr, Ni, Pb, and Fe) pollution in Lekki lagoon, Nigeria. The results revealed that intestinal nematodes contained higher levels of Cu, Cr, Ni, Pb and Fe than those recorded in fish tissues.

**Figure 3:**
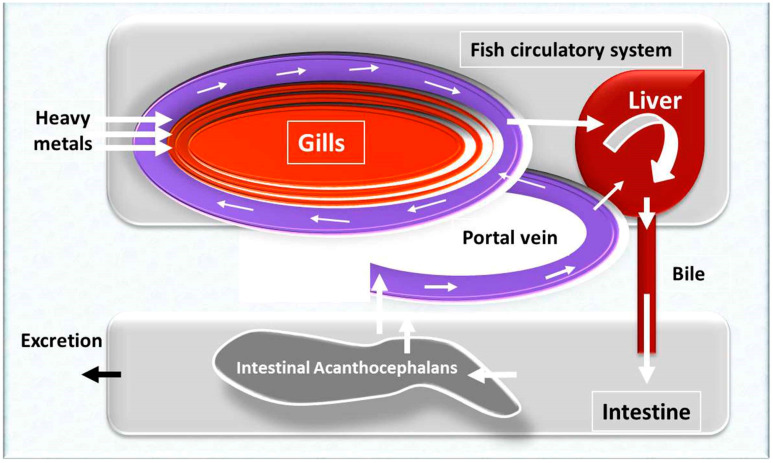
The uptake, transport and excretion of heavy metals in fish, and the route of metal uptake through the intestinal parasite such as acanthocephalans.

Recently, Hassan et al. [26] concluded that the infection of marine fish with Cestoda parasites showed a much higher bioaccumulation capacity of some heavy metals (As, Fe, Zn, Pb, Cu, Cd) than fish organs, therefore, it might act as a biological indicator for heavy metal pollution. In addition, it could also minimize the heavy metals bioaccumulation in fish tissues. Likewise, Ashmawy et al. [94] recently investigated the effect of heavy metal pollution on *Oreochromis niloticus* fish in three polluted areas in Egypt (Edku, Edfina and Mariout lake). The obtained results revealed that the increased concentration of heavy metals (lead, cadmium, and mercury) was associated with the presence of Trematodes, Monogenea, Protozoa, Crustacea, and Acanthocephala. In a similar theory, the presence of *Fasciola Hepatica* and *Dicrocoelium lanceatum* in cattle significantly down-regulated the levels of copper, cadmium, lead, and zinc. Moreover, the intensity of infection was inversely correlated with the accumulation of the metal; which may indicate the possibility of metals accumulation by helminths [95]. In a more recent study, Zaki et al. [96] compared the concentration of cadmium and lead in the intestine, liver, and muscles of fish infected or not with acanthocephalan, nematodes and digenean parasites in Sharm El-Sheikh, South Sinia, Egypt. The cadmium concentrations in the intestine, liver, and muscles of non-infected and infected fish were much lower than those of lead. In addition, concentrations of both metals were decreased in the liver, intestine, and muscles of parasites-infected fish. Additionally, Hursky and Pietrock [97] found that the parasite’s bioaccumulation of selenium was low relative to its host, and parasitized trout showed low muscle Se accumulation as compared to uninfected fish. The uptake, transport, and excretion of heavy metals in fish and the route of metal uptake through the intestinal parasite such as acanthocephalans are illustrated in Figure 3. Additionally, the findings of some recent studies using parasites as biological indicators for heavy metal bioaccumulation are presented in Table 3.

## 5. Conclusions and Future Perspectives

Fish are the most important aquatic organisms that can accumulate heavy metals in their organs. Heavy metals induce significant damage to the physiologic and biochemical processes of the fish and subsequently to fish consumers. There is a strong relationship between heavy metal pollution and parasites, whereas the parasite enhances the toxic effects of heavy metals by interfering with fish-protection mechanisms, inducing negative effects on the physiological homeostasis of fish. However, parasitic infestation in fish may have some profits, where some types of intestinal parasites (such as acanthocephalan) can bioaccumulate heavy metals in their tissues several thousand times higher than the fish tissues. The presence of the bile acids in the lumen of fish leads to the formation of organic-metallic complexes, which are easily absorbed by the worms due to lipophilicity. By using parasitism and heavy metals as a bioindicator for pollution of the fish ecosystem, we can detect the health status of the fish environment. Additionally, we found that the parasitism had an environmental impact and demonstrated that parasites are important for biodiversity and development, as well as a healthy system which is rich in a diverse parasite fauna. Consequently, the parasite might be used as a good biological indicator for the environmental quality of the ecosystem. 

## Figures and Tables

**Table 1:** The most common permissible limits of heavy metals in aquatic environments for fish health.

Heavy Metal	Freshwater (µg/L)	Seawater (µg/L)	References
Lead	0.18–1.00	0.02–0.05	International Lead association [42]
Mercury	0.02	0.02	Anzecc [43]
Cadmium	0.05	1.0	NWQMS [44]
Chromium	5	5	EPA [45]
Copper	0.1	2	EPA [46]
Nickel	0.1	2	NSW [47]

**Table 2:** A summary of the main findings of metal accumulation studies in different marine species. Metals were accumulated in the parasite in high ration compared to host muscle tissue.

Host	Parasite	Habitat	Ratio	Metal	Reference
**1. Acanthocephala**					
Notothenia coriiceps	*Aspersentis megarhynchus*	Antarctic	3–2210	Ag, Al, As, B, Ba, Cd, Co, Cr, Cu, Fe, Mg, Mn, Ni, Pb, Sr	Sures and Reimann [77]
The European perch (*Perca fluviatilis*, L.)	*Acanthocephalus lucii*	Ružín reservoir in eastern Slovakia		As, Cd, Cr, Cu, Hg, Mn, Ni, Pb, and Zn	Brázová et al. [78]
**2. Cestoda**					
*Carcharhinus dussumieri*	*Paraorigmatobothrium* spp.	Gulf Persian	394–458	Cd, Pb	Malek et al. [79]
*Himantura cf. gerrardi*	*Polypocephalus* spp.	Gulf of Oman	5–6	Cd, Pb	Golestaninasab et al. [80]
	*Tetragonocephalum* spp.		1.5–2		
	*Rhinebothrium* spp. 1		1.2–2.5		
*Glaucostegus granulatus*	*Rhinebothrium* spp. 2		2.4–3.7		
**3. Nematoda**					
*Scophthalmus maximus*	*Bothriocephalus scorpii*	Baltic Sea	1–60	Cd, Pb	Sures et al. [81]
*Chasar bathybius*	*Dichelyne minutus*	Caspian Sea	19–194	Cu, Zn	Amini et al. [10]
*Trichiurus leptarus*	*Hysterothylacium* spp.	Gulf of Oman	8–790	Cd, Pb	Khaleghzadeh-Ahangar et al. [24]
*Sparus aurata*	*Hysterothylacium aduncum*	Mediterranean Sea, Turkey	1.3–53	Cd, Cr, Cu, Fe, Hg, Mn, Mg, Pb, Zn	Dural et al. [82]
*Trachurus trachurus*	*Anisakis simplex* larvae	Atlantic (Galician coast)	6–289	Cd, Cu, Pb, Zn	Pascual and Abollo [83]
*Todaropsis eblanae*			18–41		
*Dicentrarchus labrax*	*Anisakis* spp.	Mediterranean Sea, Egypt	2–16	Cd, Cu, Fe, Mn, Ni, Pb, Zn	Morsy et al. [84]
*Stenella coeruleoalba*	*Anisakis simplex* adult	Atlantic (Galician coast)	1.7–46	Cd, Cu, Pb, Zn	Pascual and Abollo [83]
*Liza vaigiensis*	*Echinocephalus* spp.	Arabian Sea	21–360	As, Cd, Fe, Hg, Pb, Zn	Azmat et al. [85]
*Liza vaigiensis*	*Ascaris* spp.		26–400		
*Globicephala melas*	*Anisakis simplex* Metal adult	Atlantic (Galician coast)	1.7–64	Cd, Cu, Pb, Zn	Pascual and Abollo [83]
*Alligator mississippiensis*	Intestinal trematodes	Florida and Louisiana		As, Cd, Cu, Fe, Pb, Se, and Zn	Tellez and Merchant [86]

**Table 3:** Findings of some recent studies using parasites as biological indicators for heavy metals bioaccumulation.

Parasite	Studied Heavy Metal/s	Host	References
Monogenea	Cr, Fe and Ni	Wild Fish	Feist and Longshaw [74]
Monogeans and Crusteacean parasites	Zn and Se	*Epinephelus tauvina, Acanthopagrus bifasciatus and Siganus rivulatus*	Bayoumy et al. [91]
Acanthocephalans	Pb	*Oreochromis niloticus*	Paller et al. [98]
*Procamallanus* spp. (intestinal nematodes)	Cu, Cr, Ni, Pb and Fe	*Parachanna obscura* and *Clarias gariepinus*	Bamidele and Kuton [93]
Acanthocephalans, larvae *Contracaecum* sp. (L3) and *Acestrorhynchus lacustris*,	Mg, Al, Ti, Cr, Mn, Fe, Ni, Cu, Zn, As, Cd, Ba, and Pb	*Acestrorhynchus lacustris*	Leite et al. [99]
*Fasciola hepatica* and *Dicrocilium lanceatum*	Cu, Cd, Pb and Zn	Cattle	Khaleghzadeh-Ahangar et al. [24]
Acanthocephalan, nematodes and digenean parasites	Cd and Pb	*Siganus rivulatus*	Al-Hasawi [100]
Liver Flukes	Cd, Cu, Pb, and Zn	Water Buffalo	Acosta et al. [101]
Wawo worms	Cd, Pb, and Hg	Marine Fish	Nachev and Sures [58]
*Procamallanus* spp. and *Siphodera ghanensis*	Pb, Zn, Mn, Fe and Cd	*S. clarias* and *C. nigrodigitatus*	Akinsanya and Kuton [102]
Anisakis parasites	Hg		Graci et al. [103]
Bacterium-like organisms and metazoan parasites	Cu, Pb and Cd	The clam (*Protothaca theca*)	Montenegro et al. [104]
geanean *gill worms*	Al, Hg, Ti, Zn, As and Mg	the Eel (*Anguilla anguilla*)	Ribeiro et al. [105]

## References

[1] Klepeis N.E., Nelson W.C., Ott W.R., Robinson J.P., Tsang A.M., Switzer P., Behar J.V., Hern S.C., Engelmann W.H. (2001). The National Human Activity Pattern Survey (NHAPS): A resource for assessing exposure to environmental pollutants. J. Expo. Sci. Environ. Epidemiol..

[2] Aladaileh S.H., Khafaga A.F., El-Hack M.E.A., Al-Gabri N.A., Abukhalil M.H., Alfwuaires M.A., Bin-Jumah M., Alkahtani S., Abdel-Daim M.M., Aleya L. (2020). Spirulina platensis ameliorates the sub chronic toxicities of lead in rabbits via anti-oxidative, anti-inflammatory, and immune stimulatory properties. Sci. Total Environ.

[3] Waheed R., El Asely A.M., Bakery H., El-Shawarby R., Abuo-Salem M., Abdel-Aleem N., Malhat F., Khafaga A., Abdeen A. (2020). Thermal stress accelerates mercury chloride toxicity in Oreochromis niloticus via up-regulation of mercury bioaccumulation and HSP70 mRNA expression. Sci. Total Environ..

[4] Sankhla M.S., Kumari M., Nandan M., Kumar R., Agrawal P. (2016). Heavy metals contamination in water and their hazardous effect on human health—A review. Int. J. Curr. Microbiol. Appl. Sci..

[5] Al-Kahtani M.A. (2009). Accumulation of heavy metals in Tilapia fish (Oreochromis niloticus) from AL-Khadoud spring, AL-Hassa, Saudi Arabia. Am. J. Appl. Sci..

[6] Gkretsi V., Mars W.M., Bowen W.C., Barua L., Yang Y., Guo L., Arnaud R.S., Dedhar S., Wu C., Michalopoulos G.K. (2007). Loss of Integrin Linked Kinase fromMouse HepatocytesIn VitroandIn VivoResults in Apoptosis and Hepatitis. Hepatology.

[7] Wakawa R., Uzairu A., Kagbu J., Balarabe M. (2008). Impact assessment of effluent discharge on physico-chemical parameters and some heavy metal concentrations in surface water of River Challawa Kano, Nigeria. Afr. J. Pure Appl. Chem..

[8] Ahmed N.F., Sadek K.M., Soliman M.K., Khalil R.H., Khafaga A.F., Ajarem J.S., Maodaa S.N., Allam A.A. (2020). Moringa Oleifera Leaf Extract Repairs the Oxidative Misbalance following Sub-Chronic Exposure to Sodium Fluoride in Nile Tilapia Oreochromis niloticus. Animals.

[9] Ayyat M.S., Ayyat A.M., Naiel M.A., Al-Sagheer A.A. (2020). Reversal effects of some safe dietary supplements on lead contaminated diet induced impaired growth and associated parameters in Nile tilapia. Aquaculture.

[10] Amini Z., Pazooki J., Abtahi B., Shokri M.R. (2013). Bioaccumulation of Zn and Cu in Chasar bathybius (Gobiidae) tissue and its nematode parasite Dichelyne minutus, southeast of the Caspian Sea, Iran. Indian J. Geomarine Sci..

[11] Sabra F.S., Mehana E. (2015). Pesticides toxicity in fish with particular reference to insecticides. Asian J. Agric. Food Sci..

[12] Baruš V., Jarkovský J., Prokeš M. (2007). Philometra ovata (Nematoda: Philometroidea): A potential sentinel species of heavy metal accumulation. Parasitol. Res..

[13] de Buron I., James E., Riggs-Gelasco P.J., Ringwood A.H., Rolando E., Richardson D. (2009). Overviewof the status of heavy metal accumulation by helminths with a note on the use of in vitro culture of adult acanthocephalans to study the mechanisms of bioaccumulation. Neotrop. Helminthol..

[14] Parmar T.K., Rawtani D., Agrawal Y. (2016). Bioindicators: The natural indicator of environmental pollution. Front. Life Sci..

[15] Vardanyan L., Schmieder K., Sayadyan H., Heege T., Heblinski J., Agyemang T., De J., Breuer J., Sengupta M., Dalwani R. (2008). Heavy metal accumulation by certain aquatic macrophytes from Lake Sevan (Armenia). Proceedings of Taal 2007, the 12th World Lake Conference: 1020–1038.

[16] Burger J. (2006). Bioindicators: A review of their use in the environmental literature 1970–2005. Environ. Bioindic..

[17] Diamant A. (1989). Ecology of the acanthocephalan Sclerocollum rubrimaris Schmidt and Paperna, 1978 (Rhadinorhynchidae: Gorgorhynchinae) from wild populations of rabbitfish (genus Siganus) in the northern Red Sea. J. Fish. Biol..

[18] Sures B. (2003). Accumulation of heavy metals by intestinal helminths in fish: An overview and perspective. Parasitology.

[19] Luckenbach T., Triebskorn R., Müller E., Oberemm A. (2001). Toxicity of waters from two streams to early life stages of brown trout (Salmo trutta f. fario L.), tested under semi-field conditions. Chemosphere.

[20] Sures B., Nachev M., Selbach C., Marcogliese D.J. (2017). Parasite responses to pollution: What we know and where we go in ‘Environmental Parasitology’. Parasites Vectors.

[21] Dural M., Göksu M.L., Özak A.A., Derici B. (2006). Bioaccumulation of some heavy metals in different tissues of Dicentrarchus labrax L, 1758, Sparus aurata L, 1758 and Mugil cephalus L, 1758 from the Camlik lagoon of the eastern cost of mediterranean (turkey). Environ. Monit. Assess..

[22] Goater T.M., Goater C.P., Esch G.W. (2014). Parasitism: The Diversity and Ecology of Animal Parasites.

[23] Najm M., Fakhar M. (2015). Helminthic parasites as heavy metal bioindicators in aquatic ecosystems. Med. Lab. J..

[24] Khaleghzadeh-Ahangar H., Malek M., McKenzie K. (2011). The parasitic nematodes Hysterothylacium sp. type MB larvae as bioindicators of lead and cadmium: A comparative study of parasite and host tissues. Parasitology.

[25] Nachev M., Schertzinger G., Sures B. (2013). Comparison of the metal accumulation capacity between the acanthocephalan Pomphorhynchus laevis and larval nematodes of the genus Eustrongylides sp. infecting barbel (Barbus barbus). Parasites Vectors.

[26] Hassan A., Moharram S., El Helaly H. (2018). Role of parasitic helminths in bioremediating some heavy metal accumulation in the tissues of Lethrinus mahsena. Turk. J. Fish. Aquat. Sci..

[27] Abollo E., Gestal C., Pascual S. (2001). Anisakis infestation in marine fish and cephalopods from Galician waters: An updated perspective. Parasitol. Res..

[28] Grutter A.S. (1999). Infestation dynamics of gnathiid isopod juveniles parasitic on the coral-reef fish Hemigymnus melapterus (Labridae). Mar. Biol..

[29] Olmedo P., Pla A., Hernández A.F., Barbier F., Ayouni L., Gil F. (2013). Determination of toxic elements (mercury, cadmium, lead, tin and arsenic) in fish and shellfish samples. Risk assessment for the consumers. Environ. Int..

[30] Khafaga A.F., El-Hack M.E.A., Taha A.E., Elnesr S.S., Alagawany M. (2019). The potential modulatory role of herbal additives against Cd toxicity in human, animal, and poultry: A review. Environ. Sci. Pollut. Res..

[31] Norouzi E., Bahramifar N., Ghasempouri S.M. (2012). Effect of teeth amalgam on mercury levels in the colostrums human milk in Lenjan. Environ. Monit. Assess..

[32] Kaur P., Aschner M., Syversen T. (2011). Biochemical factors modulating cellular neurotoxicity of methylmercury. J. Toxicol..

[33] Abadin H., Ashizawa A., Stevens Y., Llados F., Diamond G., Sage G., Citra M., Quinones A., Bosch S., Swarts S. (2007). Toxicological Profile for Lead [Internet].

[34] El-Hack M.E.A., Abdelnour S.A., El A.E.-M.E.A., Arif M., Khafaga A., Shaheen H., Samak D., Swelum A.A. (2019). Putative impacts of phytogenic additives to ameliorate lead toxicity in animal feed. Environ. Sci. Pollut. Res..

[35] Järup L. (2003). Hazards of heavy metal contamination. Br. Med. Bull..

[36] Saeed S.M., Shaker I.M. Assessment of heavy metals pollution in water and sediments and their effect on Oreochromis niloticus in the northern delta lakes, Egypt. Proceedings of the 8th International Symposium on Tilapia in Aquaculture.

[37] Haseena M., Malik M.F., Javed A., Arshad S., Asif N., Zulfiqar S., Hanif J. (2017). Water pollution and human health. Environ. Risk Assess. Remediat..

[38] Anyanwu B.O., Ezejiofor A.N., Igweze Z.N., Orisakwe O.E. (2018). Heavy metal mixture exposure and effects in developing nations: An update. Toxics.

[39] Afshan S., Ali S., Ameen U.S., Farid M., Bharwana S.A., Hannan F., Ahmad R. (2014). Effect of different heavy metal pollution on fish. Res. J. Chem. Environ. Sci..

[40] Padrilah S.N., Shukor M.Y.A., Yasid N.A., Ahmad S.A., Sabullah M.K., Shamaan N.A. (2018). Toxicity Effects of Fish Histopathology on Copper Accumulation. Pertanika J. Trop. Agric. Sci..

[41] Cobbina S.J., Xu H., Zhao T., Mao G., Zhou Z., Wu X., Liu H., Zou Y., Wu X., Yang L. (2015). A multivariate assessment of innate immune-related gene expressions due to exposure to low concentration individual and mixtures of four kinds of heavy metals on zebrafish (Danio rerio) embryos. Fish. Shellfish Immunol..

[42] Association I.L. (2017). Lead in Aquatic Environments. Understanding the Science.

[43] Anzecc A. (2000). Australian and New Zealand guidelines for fresh and marine water quality. Aust. N. Z. Environ. Conserv. Counc. Agric. Resour. Manag. Counc. Aust. N. Z. Canberra.

[44] National Water Quality Management Strategy Aquatic Ecosystems Rationale and Background Information (Chapter 8). https://www.waterquality.gov.au/sites/default/files/documents/anzecc-armcanz-2000-guidelines-vol2.pdf.

[45] NSW EPA (2000). State of the Environment 2000. Syd. Environ. Prot. Auth. NSW Aust..

[46] EPA NSW (2000). State of the Environment Report. NSW Environ. Prot. Auth..

[47] EPA NSW (2000). State of the Environment Report. Environ. Prot. Auth. NSW.

[48] Fatima M., Usmani N., Hossain M.M. (2014). Heavy metal in aquatic ecosystem emphasizing its effect on tissue bioaccumulation and histopathology: A review. J. Environ. Sci. Technol..

[49] Jan A., Azam M., Siddiqui K., Ali A., Choi I., Haq Q. (2015). Heavy metals and human health: Mechanistic insight into toxicity and counter defense system of antioxidants. Int. J. Mol. Sci..

[50] Naiel M.A., Ismael N.E., Abd El-hameed S.A., Amer M.S. (2020). The antioxidative and immunity roles of chitosan nanoparticle and vitamin C-supplemented diets against imidacloprid toxicity on Oreochromis niloticus. Aquaculture.

[51] Ezzat S., ElKorashey R., Sherif M. (2012). The economical value of nile tilapia fish Oreochromis niloticus in relation to water quality of Lake Nasser, Egypt. J. Am. Sci..

[52] Riad S., Safaa H., Mohamed F. (2010). Influence of probiotic, prebiotic and/or yeast supplementation in broiler diets on the productivity, immune response and slaughter traits. J. Ani. Poult..

[53] Al Naggar Y., Khalil M.S., Ghorab M.A. (2018). Environmental pollution by heavy metals in the aquatic ecosystems of Egypt. Open Acc. J. Toxicol.

[54] Zeitoun M.M., Mehana E. (2014). Impact of water pollution with heavy metals on fish health: Overview and updates. Glob. Vet..

[55] Atobatele O.E., Olutona G.O. (2015). Distribution of three non-essential trace metals (Cadmium, Mercury and Lead) in the organs of fish from Aiba Reservoir, Iwo, Nigeria. Toxicol. Rep..

[56] Nwabunike M. (2016). The effects of bioaccumulation of heavy metals on fish fin over two years. J. Fish. Livest. Prod..

[57] Rajeshkumar S., Li X. (2018). Bioaccumulation of heavy metals in fish species from the Meiliang Bay, Taihu Lake, China. Toxicol. Rep..

[58] Nachev M., Sures B. (2016). Environmental parasitology: Parasites as accumulation bioindicators in the marine environment. J. Sea Res..

[59] Le T.Y., Nachev M., Grabner D., Hendriks A.J., Sures B. (2016). Development and validation of a biodynamic model for mechanistically predicting metal accumulation in fish-parasite systems. PLoS ONE.

[60] Oumar D.A., Flibert G., Tidjani A., Rirabe N., Patcha M., Bakary T., Ousman A.H., Yves T., Aly S. (2018). Risks Assessments of Heavy Metals Bioaccumulation in Water and Tilapia nilotica Fish from Maguite Island of Fitri Lake. Curr. J. Appl. Sci. Technol..

[61] Eissa O.S. (2004). Protective effect of vitamin C and glutathione against the histopathological changes induced by imidacloprid in the liver and testis of Japanese quail. Egypt. J. Hosp. Med..

[62] Kim H.-T., Loftus J.P., Mann S., Wakshlag J.J. (2018). Evaluation of Arsenic, Cadmium, Lead and Mercury Contamination in Over-the-Counter Available Dry Dog Foods With Different Animal Ingredients (Red Meat, Poultry, and Fish). Front. Vet. Sci..

[63] Mirghaed A.T., Hoseini S.M., Ghelichpour M. (2018). Effects of dietary 1, 8-cineole supplementation on physiological, immunological and antioxidant responses to crowding stress in rainbow trout (Oncorhynchus mykiss). Fish. Shellfish Immunol..

[64] Naiel M.A., Ismael N.E., Shehata S.A. (2019). Ameliorative effect of diets supplemented with rosemary (Rosmarinus officinalis) on aflatoxin B1 toxicity in terms of the performance, liver histopathology, immunity and antioxidant activity of Nile Tilapia (Oreochromis niloticus). Aquaculture.

[65] Abalaka S.E. (2015). Heavy metals bioaccumulation and histopathological changes in Auchenoglanis occidentalis fish from Tiga dam, Nigeria. J. Environ. Health Sci. Eng..

[66] Saini A.K. (2012). Toxic Effects on Fish Inhabiting Arsenic Contaminated Fresh Waters of Punjab. Ph.D. Thesis.

[67] Deore S., Wagh S. (2012). Heavy metal induced histopathological alterations in liver of Channa gachua (Ham). J. Exp. Sci..

[68] Dhevakrishnan R., Zaman G. (2012). Cauvery river pollutants induced histopathological changes in kidney and muscle tissues of freshwater fish, Labio rohita (Hamilton, 1822). Online Int. Interdiscip. Res. J..

[69] Fernández-Rubio C., Ordonez C., Abad-González J., Garcia-Gallego A., Honrubia M.P., Mallo J.J., Balana-Fouce R. (2009). Butyric acid-based feed additives help protect broiler chickens from Salmonella Enteritidis infection. Poult. Sci..

[70] Mshelbwala F.M., Ibrahim N.D.-G., Saidu S.N., Azeez A.A., Akinduti P.A., Kwanashie C.N., Kadiri A.K.F., Muhammed M., Fagbamila I.O., Luka P.D. (2017). Motile Salmonella serotypes causing high mortality in poultry farms in three South-Western States of Nigeria. Vet. Rec. Open.

[71] Misganaw K., Getu A. (2016). Review on major parasitic crustacean in fish. Fish. Aquac. J..

[72] Poulin R. (2007). Evolutionary Ecology of Parasites.

[73] Palm H.W. (2011). Fish parasites as biological indicators in a changing world: Can we monitor environmental impact and climate change?. Progress in Parasitology.

[74] Feist S., Longshaw M. (2008). Histopathology of fish parasite infections–importance for populations. J. Fish. Biol..

[75] Mohammadi F., Mousavi S.M., Rezaie A. (2012). Histopathological study of parasitic infestation of skin and gill on Oscar (Astronotus ocellatus) and discus (Symphysodon discus). Aquac. Aquar. Conserv. Legis..

[76] Nahavandinejad M., Seidavi A., Asadpour L., Payan-Carreira R. (2014). Blood biochemical parameters of broilers fed differently thermal processed soybean meal. Rev. Mvz Córdoba.

[77] Sures B., Reimann N. (2003). Analysis of trace metals in the Antarctic host-parasite system Notothenia coriiceps and Aspersentis megarhynchus (Acanthocephala) caught at King George Island, South Shetland Islands. Polar Biol..

[78] Brázová T., Hanzelová V., Miklisová D., Šalamún P., Vidal-Martínez V.M. (2015). Host-parasite relationships as determinants of heavy metal concentrations in perch (Perca fluviatilis) and its intestinal parasite infection. Ecotoxicol. Environ. Saf..

[79] Malek M., Haseli M., Mobedi I., Ganjali M., Mackenzie K. (2007). Parasites as heavy metal bioindicators in the shark Carcharhinus dussumieri from the Persian Gulf. Parasitology.

[80] Golestaninasab M., Malek M., Roohi A., Karbassi A., Amoozadeh E., Rashidinejad R., Ghayoumi R., Sures B. (2014). A survey on bioconcentration capacities of some marine parasitic and free-living organisms in the Gulf of Oman. Ecol. Indic..

[81] Sures B., Taraschewski H., Rokicki J. (1997). Lead and cadmium content of two cestodes, Monobothrium wageneri and Bothriocephalus scorpii, and their fish hosts. Parasitol. Res..

[82] Dural M., Genc E., Sangun M.K., Güner Ö. (2011). Accumulation of some heavy metals in Hysterothylacium aduncum (Nematoda) and its host sea bream, Sparus aurata (Sparidae) from North-Eastern Mediterranean Sea (Iskenderun Bay). Environ. Monit. Assess..

[83] Pascual S., Abollo E. (2005). Whaleworms as a tag to map zones of heavy-metal pollution. Trends Parasitol..

[84] Morsy K., Bashtar A.-R., Abdel-Ghaffar F., Mehlhorn H., Al Quraishy S., El-Mahdi M., Al-Ghamdi A., Mostafa N. (2012). First record of anisakid juveniles (Nematoda) in the European seabass Dicentrarchus labrax (family: Moronidae), and their role as bio-indicators of heavy metal pollution. Parasitol. Res..

[85] Azmat R., Fayyaz S., Kazi N., Mahmood S.J., Uddin F. (2008). Natural bioremediation of heavy metals through nematode parasite of fish. Biotechnology.

[86] Tellez M., Merchant M. (2015). Biomonitoring heavy metal pollution using an aquatic apex predator, the American alligator, and its parasites. PLoS ONE.

[87] Sures B. (2001). The use of fish parasites as bioindicators of heavy metals in aquatic ecosystems: A review. Aquat. Ecol..

[88] Sures B., Siddall R., Taraschewski H. (1999). Parasites as accumulation indicators of heavy metal pollution. Parasitol. Today.

[89] Sures B. (2008). Host–parasite interactions in polluted environments. J. Fish. Biol..

[90] Shafi N., Ayub J., Akhtar T. (2015). Physico-chemical variables and fish parasites of River Neelum Azad Jammu and Kashmir, Pakistan. J. Bioresour. Manag..

[91] Bayoumy E.M., Abou-El-dobal S.K., Hassanain M.A. (2015). Assessment of Heavy Metal Pollution and Fish Parasites as Biological Indicators at Arabian Gulf off Dammam Coast, Saudi Arabia. Int. J. Zool. Res..

[92] Gilbert B.M., Avenant-Oldewage A. (2017). Parasites and pollution: The effectiveness of tiny organisms in assessing the quality of aquatic ecosystems, with a focus on Africa. Environ. Sci. Pollut. Res..

[93] Bamidele A., Kuton M.P. (2016). Parasitic diseases and heavy metal analysis in Parachanna obscura (Gunther 1861) and Clarias gariepinus (Burchell 1901) from Epe Lagoon, Lagos, Nigeria. Asian Pac. J. Trop. Dis..

[94] Ashmawy K.I., Hiekal F.A., Abo-Akadda S.S., Laban N.E. (2018). The inter-relationship of water quality parameters and fish parasite occurrence. Alex. J. Vet. Sci..

[95] Khovidhunkit W., Kim M.-S., Memon R.A., Shigenaga J.K., Moser A.H., Feingold K.R., Grunfeld C. (2004). Effects of infection and inflammation on lipid and lipoprotein metabolism: Mechanisms and consequences to the host. J. Lipid Res..

[96] Zaki M., Salem M.E.-S., Gaber M., Nour A. (2015). Effect of chitosan supplemented diet on survival, growth, feed utilization, body composition & histology of Sea bass (Dicentrarchus labrax). World, J. Eng. Technol..

[97] Hursky O., Pietrock M. (2015). Intestinal nematodes affect selenium bioaccumulation, oxidative stress biomarkers, and health parameters in juvenile rainbow trout (Oncorhynchus mykiss). Environ. Sci. Technol..

[98] Paller V.G.V., Resurreccion D.J.B., de la Cruz C.P.P., Bandal M.Z. (2016). Acanthocephalan parasites (Acanthogyrus sp.) of Nile tilapia (Oreochromis niloticus) as biosink of lead (Pb) contamination in a Philippine freshwater lake. Bull. Environ. Contam. Toxicol..

[99] Leite L.A., Pedro N.H., de Azevedo R.K., Kinoshita A., Gennari R.F., Watanabe S., Abdallah V.D. (2017). Contracaecum sp. parasitizing Acestrorhynchus lacustris as a bioindicator for metal pollution in the Batalha River, southeast Brazil. Sci. Total Environ..

[100] Al-Hasawi Z.M. (2019). Environmental Parasitology: Intestinal helminth parasites of the siganid fish Siganus rivulatus as bioindicators for trace metal pollution in the Red Sea. Parasite.

[101] Acosta A.G.D., Camara C.N.M., Ongsiako J.R.M.J., Tsoi J.N., Flores M.J.C., Janairo J.I.B. (2017). Bioaccumulation of Cadmium, Copper, Lead, and Zinc in Water Buffaloes (Bubalusbubalis) Infected with Liver Flukes (Fasciolagigantica). Orient. J. Chem..

[102] Akinsanya B., Kuton M.P. (2016). Bioaccumulation of heavy metals and parasitic fauna in Synodontis clarias (Linnaeus, 1758) and Chrysichthys nigrodigitatus (Lacepede, 1803) from Lekki Lagoon, Lagos, Nigeria. Asian Pac. J. Trop. Dis..

[103] Graci S., Collura R., Cammilleri G., Buscemi M.D., Giangrosso G., Principato D., Gervasi T., Cicero N., Ferrantelli V. (2017). Mercury accumulation in Mediterranean Fish and Cephalopods Species of Sicilian coasts: Correlation between pollution and the presence of Anisakis parasites. Nat. Prod. Res..

[104] Montenegro D., Valdés J., González M.T. (2019). Histopathological lesions, pathogens and parasites as health indicators of an edible clam (Protothaca thaca) inhabiting a bay exposed to anthropogenic activities in Northern Chile. Environ. Monit. Assess..

[105] Ribeiro C.O., Vollaire Y., Sanchez-Chardi A., Roche H. (2005). Bioaccumulation and the effects of organochlorine pesticides, PAH and heavy metals in the Eel (Anguilla anguilla) at the Camargue Nature Reserve, France. Aquat. Toxicol..

